# The application of the ICD-10 for antepartum stillbirth patients in a referral centre of Eastern China: a retrospective study from 2015 to 2022

**DOI:** 10.1186/s12884-024-06313-5

**Published:** 2024-02-26

**Authors:** Chuan-Shou Feng, Shu-Fen Li, Hui-Hui Ju

**Affiliations:** Obstetrical department, Changzhou Women and Children Health Hospital, Nanjing Medical University, Changzhou, Jiangsu China

**Keywords:** International classification of diseases, Antepartum stillbirth, Causes of stillbirth, Retrospective study, Autopsy, Chromosomal microarray analysis

## Abstract

**Background:**

The causes of some stillbirths are unclear, and additional work must be done to investigate the risk factors for stillbirths.

**Objective:**

To apply the International Classification of Disease-10 (ICD-10) for antepartum stillbirth at a referral center in eastern China.

**Methods:**

Antepartum stillbirths were grouped according to the cause of death according to the International Classification of Disease-10 (ICD-10) criteria. The main maternal condition at the time of antepartum stillbirth was assigned to each patient.

**Results:**

Antepartum stillbirths were mostly classified as fetal deaths of unspecified cause, antepartum hypoxia. Although more than half of the mothers were without an identified condition at the time of the antepartum stillbirth, where there was a maternal condition associated with perinatal death, maternal medical and surgical conditions and maternal complications during pregnancy were most common. Of all the stillbirths, 51.2% occurred between 28 and 37 weeks of gestation, the main causes of stillbirth at different gestational ages also differed. Autopsy and chromosomal microarray analysis (CMA) were recommended in all stillbirths, but only 3.6% received autopsy and 10.5% underwent chromosomal microarray analysis.

**Conclusions:**

The ICD-10 is helpful in classifying the causes of stillbirths, but more than half of the stillbirths in our study were unexplained; therefore, additional work must be done. And the ICD-10 score may need to be improved, such as by classifying stillbirths according to gestational age. Autopsy and CMA could help determine the cause of stillbirth, but the acceptance of these methods is currently low.

**Supplementary Information:**

The online version contains supplementary material available at 10.1186/s12884-024-06313-5.

## Background

Stillbirth is usually defined as death after 20 weeks of pregnancy in most developed countries and after 28 weeks of pregnancy in developing countries, before complete expulsion or extraction from the mother of a product of conception, which is indicated by the fact that after such separation, the fetus does not show any evidence of life [1, 2]. In China, stillbirth is defined as the death of a fetus after 20 weeks of gestation.

Stillbirth is a serious adverse pregnancy outcome and a common global public health problem. The global stillbirth rate is estimated to be 18·4 per 1000 births [3]. Globally, each year, approximately 2·6 million stillbirths occur, 99% of which occur in low- and middle-income countries (LMICs) [3–6]. Studies have shown that stillborn infants are more likely to be antepartum, with only a few of the deaths occurring intrapartum. In United Kingdom, 48·3% of stillbirths occur during the antepartum period [7]. Irisa Zile et al. [8] reported that 73.5% of stillborn neonates were antepartum. In 2016, approximately 90% of cases occurred before labor started in Sweden [9]. Based on the Every Newborn Action Plan to improve newborn health and prevent stillbirths, a stillbirth target of 12 or less stillbirths per 1000 total births for all countries by 2030 was set, with a focus on addressing inequalities and the use of audit data to track and prevent stillbirths [10].

However, current researches on the causes of stillbirths are insufficient, and many stillbirths are of unknown cause, especially there is little research published on differences in maternal and fetal characteristics associated with antepartum stillbirth. So, more work must be done to investigate the risk factors for stillbirths, to determine which are preventable, and to provide the right advice to parents after stillbirths and help them build their future pregnancy plans.

The classification system helps to divide the causes of stillbirth into relevant groups to assist in counselling and the development of family planning. A number of classification systems have been applied to stillbirth in different countries [11, 12], however, global comparisons are difficult because of the multiple classification systems used for perinatal death [12, 13]. Better classification systems are needed to achieve accuracy and consistency in the reporting of causes of stillbirths.

The World Health Organization adapted the existing International Classification of Diseases, tenth revision (ICD-10), for perinatal death as a globally applicable and comparable system in 2016 [14], the new International Classification of Diseases for Perinatal Mortality (ICD-PM) classification system uses stratification to further determine the causes of fetal death and/or contributing maternal conditions. The ICD-PM has three distinct features. It identifies the timing of perinatal death (antepartum, intrapartum, neonatal); the causes of death linked to existing ICD codes are logically grouped; and ICD-PM links the maternal condition with the perinatal death. This new classification system will contribute to more accurate and uniform reporting for comparison in various situations.

In this study, we evaluated the current status of antepartum stillbirths in a referral center of eastern China using the International Classification of Diseases (ICD-10) to classify the causes of stillbirths and determine relevant preventive measures. At the same time, the frequency of autopsy and chromosomal microarray analysis in stillbirth cases was investigated.

## Methods

This retrospective study was conducted at Changzhou Women and Children Health Hospital affiliated to Nanjing Medical University. Changzhou is a city of more than 5 million people in China’s developed eastern coastal region, and our hospital is the only tertiary hospital of obstetrics and gynecology; it is the only regional high-risk maternal treatment center and prenatal diagnosis center in the region, with 1,000 beds, and in 2022, 9896 deliveries.

All patients with antepartum stillbirths at Changzhou Women and Children Health Hospital affiliated to Nanjing Medical University from January 2015 through December 2022 were included in this study. Antepartum stillbirths were defined as fetal death occurring after 20 completed weeks of gestation. or birthweight ≥ 350 g if gestational age is unknown.

The data were extracted from outpatient obstetric examination records, hospital admissions and delivery registers. The placentas of all antepartum stillbirths were routinely pathologically examined, autopsies and chromosomal microarray analysis (CMA) were recommended for all patients. If the parents refused, the reasons for refusal were inquired in detail and recorded. Stillbirths are serious complications in obstetrics, and we attach great importance to every case of stillbirth. So, in our hospital, it is routine to discuss every case of stillbirth to find the cause of stillbirth as much as possible. Multi-disciplinary meetings with doctors, nurses, and midwives from the hospital were conducted to identify the most likely cause of fetal death as well as other contributing maternal conditions via consensus. The causes of antepartum stillbirths were analyzed with respect to clinical information and classified according to ICD-10. Antepartum stillbirths were further classified into the six ICD-PM sub-categories (A1 to A5, with A6 representing cause unknown). The contributing maternal conditions were classified into five major categories (M1 to M4, with M5 representing the unknown cases) [14] (Table 1). Gestational age was determined mainly by the final menstrual period or ultrasound results in early pregnancy if the gestational age did not match.

**Table 1:** ICD-PM categories with description and exemplar-specific causes

	Category	Description	Examples
Antepartum stillbirths	A1	Congenital malformations and chromosomal abnormalities	Anencephaly, encephalocele, microcephaly, congenital hydrocephalus, spina bifida, etc.
	A2	Infection	Congenital syphilis, congenital malaria, congenital rubella syndrome, congenital TB, etc.
	A3	Antepartum hypoxia	Intrauterine hypoxia.
	A4	Other specified antepartum disorder	Vasa previa, ruptured cord, twin-twin transfusion, Intraventricular (nontraumatic) haemorrhage, Rhesus and ABO isoimmunization, etc.
	A5	Disorders related to fetal growth	Small for gestational age, macrosomia, post-term, etc.
	A6	Antepartum death of unspecified cause	Intrauterine death of unspecified cause
Maternal conditions	M1	Complications of placenta, cord and membranes	Abruptio placentae, prolapsed cord, chorioamnionitis, etc.
	M2	Maternal complications of pregnancy	Premature rupture of membranes, oligo- and polyhydramnios, ectopic pregnancy, multiple pregnancy, etc.
	M3	Other complications of labour and delivery	Breech delivery and extraction, forceps delivery, Caesarean delivery.
	M4	Maternal medical conditions	hypertensive disorders, maternal injury, maternal use of tobacco, alcohol or drugs, etc.
	M5	No maternal conditions	No condition identified.

10.1371/journal.pone.0215864.t001

### Data analysis

Descriptive analyses were conducted using SPSS. Simple statistical tests using absolute numbers were used to calculate percentages.

## Results

From January 2015 through December 2022, a total of 87,588 women gave birth in Changzhou Women and Children Health Hospital affiliated to Nanjing Medical University, of which we reviewed data on a total of 420 (0.48%) antepartum stillbirths. Table 2 maps the demographic and clinical characteristics of women who experienced a stillbirth. Among the patients, 248 (59·0%) were primipara and 172 (41·0%) were multigravida. In addition, 173 (41.2%) were from urban areas and 247 (58.8%) from rural areas. Data on maternal ages and gestational ages were tested to have a normal distribution. The average age of the women who had stillbirth were 28·99 ± 5·38 (17–44) years, with a median of 28 years, and the average gestational age were 30.06 ± 5·74 (20–41) weeks, with a median was 30 weeks.

**Table 2:** Demographic and clinical characteristics of women who experienced a stillbirth at the Changzhou Women and Children Health Hospital, 2015–2022

Characteristics		2015 *n* = 125%)	2016 *n* = 75(%)	2017 *n* = 69(%)	2018 *n* = 33(%)	2019 *n* = 47(%)	2020 *n* = 25(%)	2021 *n* = 23(%)	2022 *n* = 23(%)	Total *n* = 420(%)
**Maternal age** **(years)**	< 18	2(1.6)	0(0。0)	0(0.0)	0(0.0)	0(0.0)	0(0.0)	0(0.0)	1(4.4)	3(0.7)
	18–35	105(84.0)	62(82.7)	61(88.4)	28(84.8)	43(91.5)	22(88.0)	18(78.3)	19(82.6)	358(85.2)
	> 35	18(14.4)	13(17.3)	8(11.6)	5(15.2)	4(8.5)	3(12.0)	5(21.7)	3(13.0)	59(14.1)
**Gestational age** **at birth**	< 28	49(39.2)	34(45.3)	28(40.6)	11(33.3)	16(34.1)	0(0.0)	0(0.0)	0(0.0)	138(32.8)
	28–37	59(47.2)	29(38.7)	30(43.5)	20(60.6)	26(55.3)	17(68.0)	17(73.9)	17(73.9)	215(51.2)
	> 37	17(13.6)	12(16.0)	11(15.9)	2(6.1)	5(10.6)	8(32.0)	6(26.1)	6(26.1)	67(16.0)
**Parity**	Primipara	79(63.2)	41(54.7)	41(59.4)	18(54.5)	30(63.8)	11(44.0)	11(47.8)	16(69.6)	248(59.0)
	Multipara	46(36.8)	34(45.3)	28(50.6)	15(45.5)	17(36.2)	14(56.0)	12(52.2)	7(30.4)	172(41.0)
**Regular check-ups**	yes	37(29.6)	35(46.7)	33(47.8)	17(51.5)	25(53.2)	17(68.0)	16(69.6)	18(78.3)	198(47.1)
	no	88(70.4)	40(53.3)	36(52.2)	16(48.5)	22(46.8)	8(32.0)	7(30.4)	5(21.7)	222(52.9)
**Residence**	Urban areas	43(34.4)	19(25.3)	19(27.5)	16(48.5)	21(44.7)	17(68.0)	19(82.6)	19(82.6)	173(41.2)
	Rural areas	82(65.6)	56(74.7)	50(72.5)	17(51.5)	26(55.3)	8(32.0)	4(17.4)	4(17.4)	247(58.8)
**Type of** **Pregnancy**	Singleton	122(97.6)	71(94.7)	66(95.7)	33(100.0)	46(97.9)	24(96.0)	23(100.0)	23(100.0)	408(97.1)
	Multiple	3(2.4)	4(5.3)	3(4.3)	0(0.0)	1(2.1)	1(4.0)	0(0.0)	0(0.0)	12(2.9)
**BMI (kg/m** ^**2**^ **)**	< 28	103(82.4)	58(77.3)	47(68.1)	26(78.8)	35(74.5)	19(76)	19(82.6)	17(73.9)	324(77.1)
	≥ 28	22(17.6)	17(22.7)	22(31.9)	7(21.2)	12(25.5)	6(24)	4(17.4)	6(26.1)	96(22.9)
**Fetal sex**	Male	61(48.8)	39(52)	37(53.6)	14(42.4)	22(46.8)	14(56)	11(47.8)	10(43.5)	208(49.5)
	Female	64(51.2)	36(48)	32(46.4)	19(57.6)	25(53.2)	11(44)	12(52.2)	13(56.5)	212(50.5)
**Autopsy**	yes	1(0.8)	1(1.3)	2(2.9)	1(3.0)	3(6.4)	1(4.0)	3(13.0)	3(13.0)	15(3.6)
	no	124(99.2)	74(98.7)	67(97.1)	32(97.0)	44(93.6)	24(96.0)	20(87.0)	20(87.0)	405(96.4)
**CMA**	yes	0(0.0)	5(6.7)	3(4.3)	6(18.2)	12(25.5)	6(24.0)	5(21.7)	7(30.4)	44(10.5)
	no	125(100.0)	70(93.3)	66(95.7)	27(81.8)	35(74.5)	19(76.0)	18(78.3)	16(69.6)	376(89.5)

Table 3 maps the causes of stillbirth against the maternal conditions for all antepartum stillbirths using the ICD-PM. Antepartum stillbirths were mostly classified as fetal deaths of unspecified causes (*n* = 235, 56.0%), fetal anomalies and chromosomal abnormalities (*n* = 49, 11.7%), or other specified antepartum disorder (*n* = 45, 10.7%). In contrast, more than half (55·9%) of mothers were without an identified condition in the antepartum stillbirths, and only 44·1% of antepartum deaths could be classified into one of the groups for associated maternal condition. M4 (Maternal medical and surgical conditions) contributed the highest proportion (*n* = 67, 16.0%).

**Table 3:** The causes of stillbirth against the maternal conditions for all antepartum stillbirths using the ICD-PM.

Maternal condition	M1	M2	M3	M4	M5	Total (%)
Antepartum stillbirths						
A1	11	4	10	15	9	49 (11.7)
A2	14	5	0	5	2	26 (6.2)
A3	7	6	3	16	3	35 (8.3)
A4	11	15	9	7	3	45 (10.7)
A5	3	8	3	15	1	30 (7.1)
A6	18	13	2	9	193	235 (56.0)
Total (%)	64 (15.2)	51(12.1)	27 (7.7)	67 (16.0)	211 (55.9)	420(100.0)

**Fig. 1:**
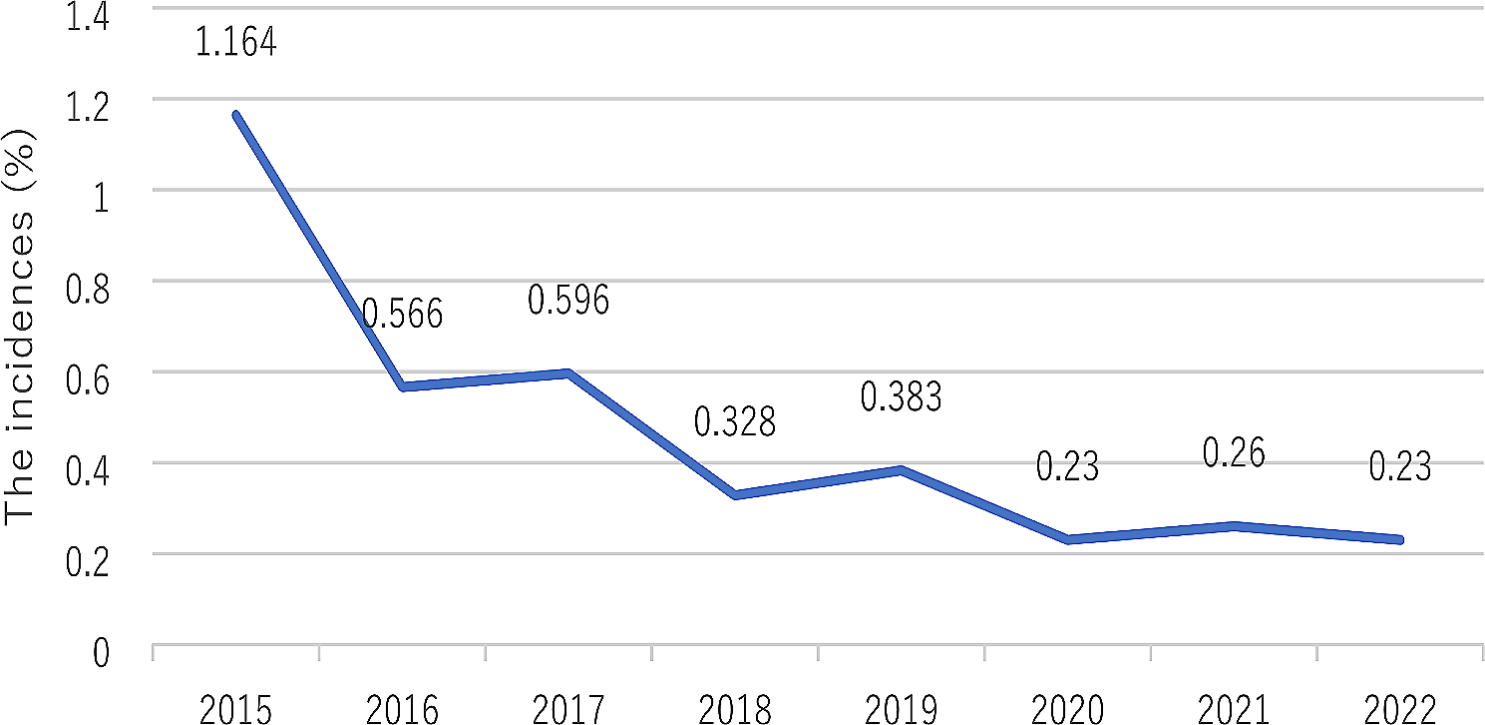
The incidences of antepartum stillbirths in the eight years (%)

**Fig. 2:**
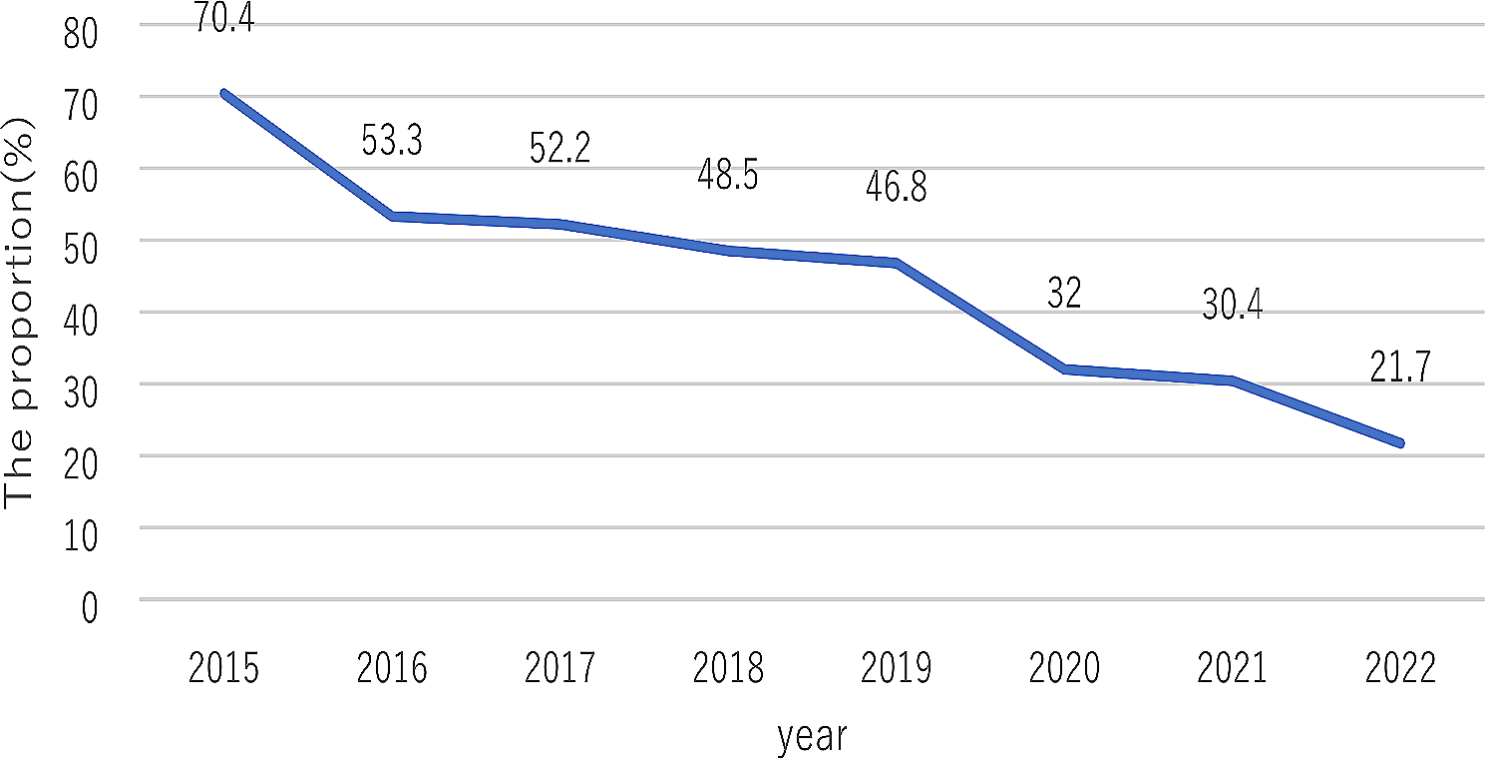
The proportion of without regular obstetric examination (%)

The annual incidence of antepartum stillbirths is shown in Fig. 1. From 2015 to 2022, there was a marked decline in the incidences of antepartum stillbirths. At the same time, the proportion of stillbirths without regular obstetric examination among all antepartum stillbirths had decrease year by year (Fig. 2). Among all the cases of stillbirths in 2015, we found that the proportion of patients without regular obstetric examination was relatively high, up to 70·4%. However, in 2022, the proportion fell to 21.7%.

**Fig. 3:**
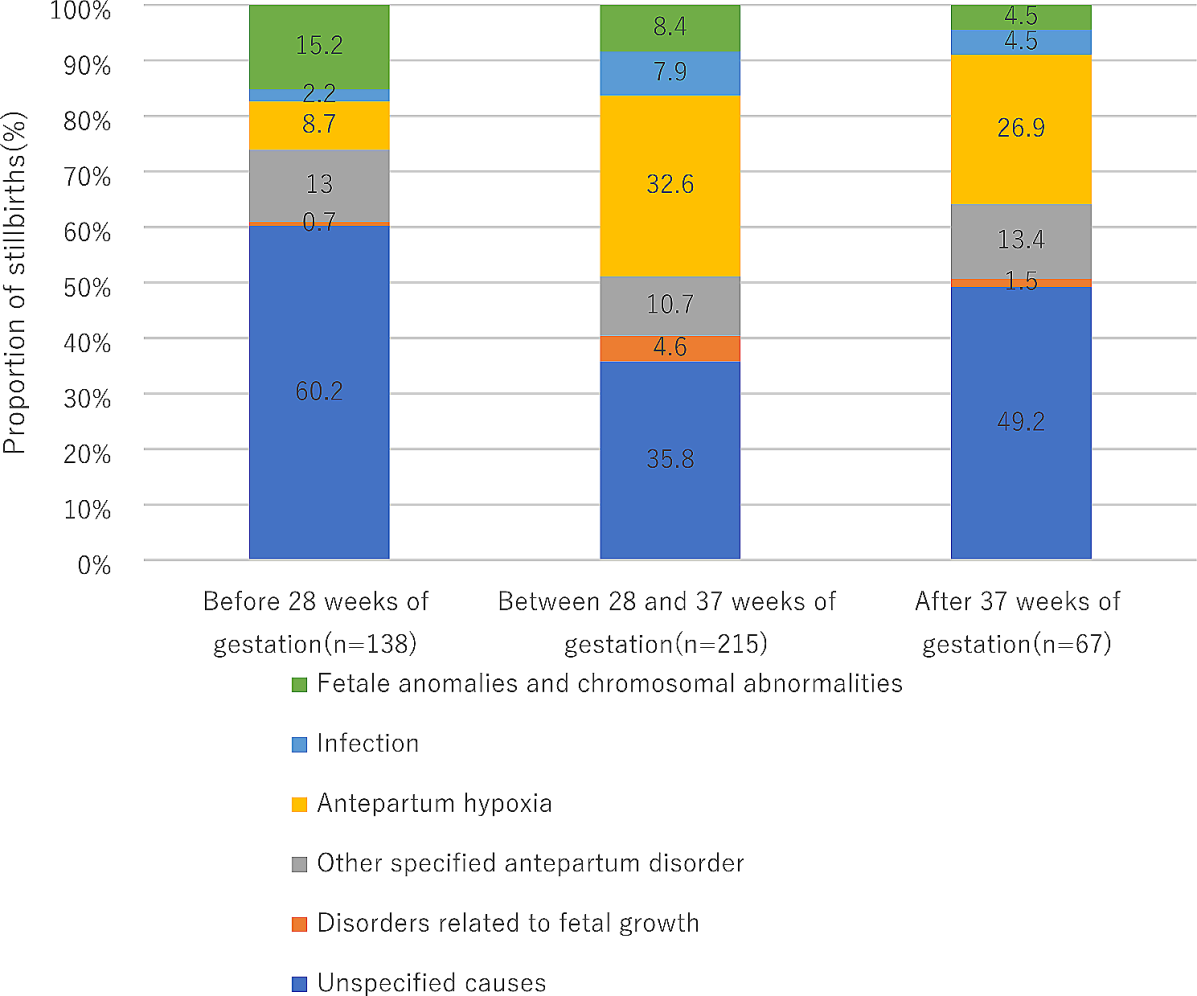
The percentage of the causes of stillbirths at different gestational ages

Figure 3 shows the percentage of causes of stillbirths by gestational age, in which more than half (51.2%) of stillbirths occurred between 28 and 37 weeks of gestation, and nearly one third occurred before 28 weeks of gestation, and only 16.0% occurred after 37 weeks of gestation. In addition, the main causes of stillbirths vary with gestational age. The main causes of stillbirths before 28 weeks of gestation were unspecified causes, fetal anomalies and chromosomal abnormalities and other specified antepartum disorder. The main causes of stillbirths between 28 and 37 weeks of gestation and after 37 weeks of gestation were all unspecified causes, antepartum hypoxia and other specified antepartum disorder.

Autopsy and chromosomal microarray analysis (CMA) were recommended for all stillbirths, but only 3.6% of patients underwent autopsy, and 10.5% underwent chromosomal microarray analysis. The autopsy of 15 patients revealed 5 abnormalities: 1 abnormal lung development, 1 cardiac malformation, 1 digestive system malformation,1 agenesis of corpus callosum, and 1 pleural effusion. Six abnormalities were found in the 44 patients according to chromosomal microarray analysis: 2 trisomy 13, 3 trisomy 18, and 1 trisomy 21.

**Fig. 4:**
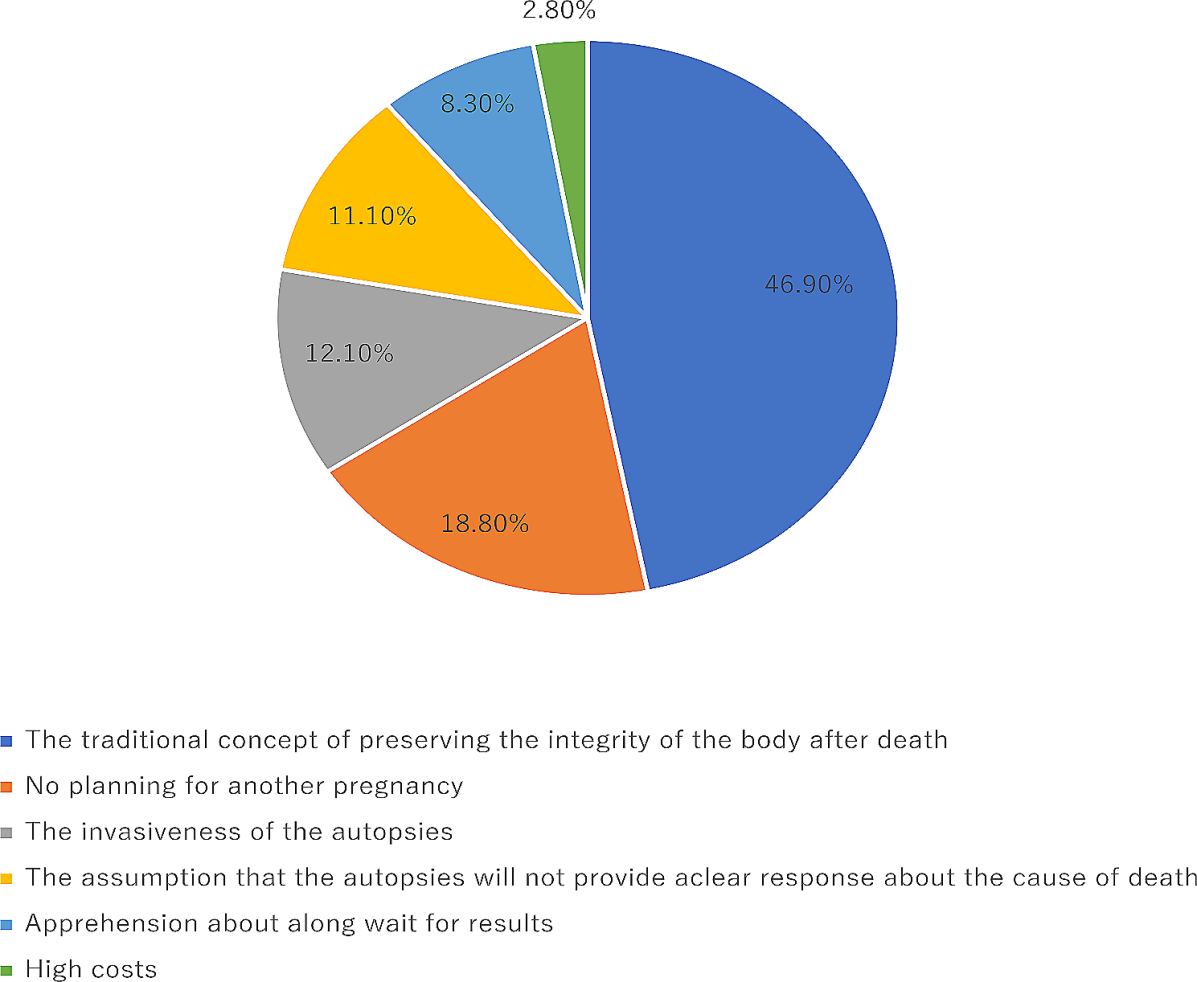
The reasons for refusing autopsies

**Fig. 5:**
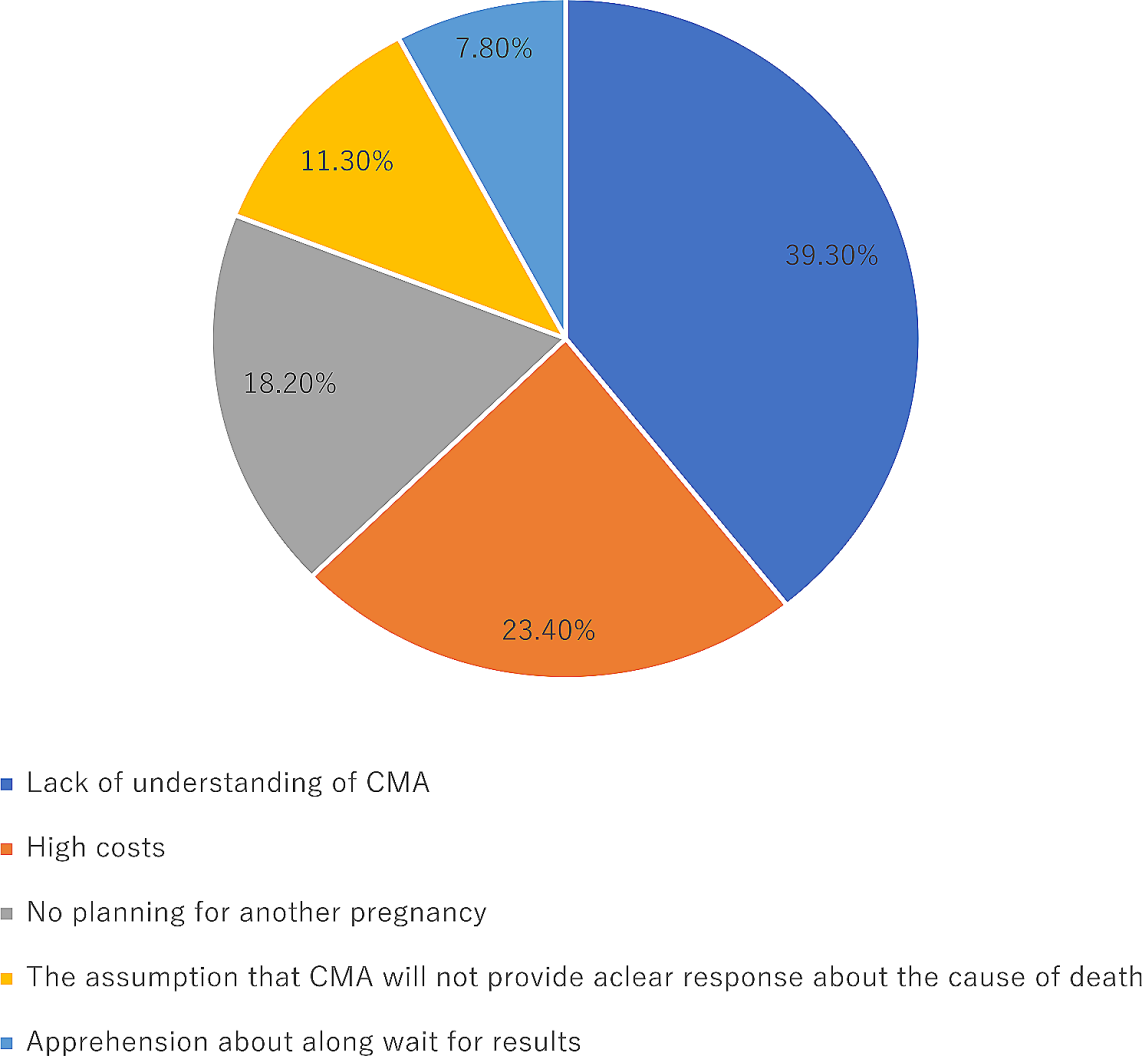
The reasons for refusing chromosomal microarray analysis

We thoroughly investigated the reasons why parents refused these two examinations. Of the 405 stillbirths in which autopsies were refused, 21 parents refused to participate in the survey, and 384 parents participated and completed the survey. The main reasons for refusing autopsies were: the traditional concept of preserving the integrity of the body after death (46·9%), no planning for another pregnancy (18·8%), the invasiveness of the autopsies (12·1%) (Fig. 4). Of the 323 stillbirths in which CMA were refused, 19 parents refused to participate in the survey, and 357 parents participated and completed the survey. The main reasons for rejecting CMA were: lack of understanding of CMA (39·3%), high costs (23·4%), no planning for another pregnancy (18·2%) (Fig. 5).

## Discussion

### Main findings

We demonstrated the application of the ICD-10 for evaluating antepartum stillbirths. In our study, we found that antepartum stillbirths were mostly classified as fetal deaths of unspecified causes, fetal anomalies and chromosomal abnormalities, or other specified antepartum disorder. More than half of these stillbirths are of unspecified cause; therefore, additional studies must be performed to address this problem. Although more than half of mothers were without an identified condition at the time of the antepartum stillbirth, where there was a maternal condition associated with perinatal deaths, maternal medical and surgical conditions and maternal complications during pregnancy were most common. Therefore, early detection of pregnancy complications and complications and standardized management and treatment were very important for reducing the incidence of stillbirth caused by these factors.

In the last eight years, the proportion of patients without regular obstetric examination has been on the decline due to the extensive publicity of the importance of obstetric examinations and the strengthening of outpatient management since 2016.

Of all the stillbirths, 32.8% occurred before 28 weeks of gestation, 51.2% occurred between 28 and 37 weeks of gestation, and 15.9% occurred after 37 weeks of gestation. The main causes of stillbirths at different gestational ages also differed. The main causes of stillbirths before 28 weeks of gestation were unspecified causes and fetal anomalies and chromosomal abnormalities. The main causes between 28 and 37 weeks of gestation were: unspecified causes and antepartum hypoxia. The main causes after 37 weeks of gestation were: unspecified causes and antepartum hypoxia. Therefore, for stillborn patients of different gestational ages, different countermeasures may need to be taken.

The main reasons for refusing autopsies were the traditional concept of preserving the integrity of the body after death, no planning for another pregnancy and the invasiveness of the autopsy. The main reasons for rejecting CMA were lack of understanding of CMA, high costs and no planning for another pregnancy.

### Strengths and limitations

In this study, we retrospectively analyzed stillbirth patients in the last 8 years, and the number of cases was relatively sufficient, which can reflect some problems to a certain extent. However, considering that this was a retrospective study, it was not conducted using the same standardized instructions, which may have affected the coding. We are unable to comment on the extent to which each prenatal stillbirth was investigated.

### Interpretation in light of previous research

Although many interventions have been implemented in many countries, stillbirths remain a major global public health problem. In many countries, stillbirths cause great pain for parents [15, 16]. Although the stillbirth rate decreased by 19.4% from 2000 to 2015 [5, 17], following various interventions worldwide, the global stillbirth rate was still as high as 18·4 per 1,000 births in 2015, or 2·6 million per year [3]. Moreover, the global stillbirth rate is extremely uneven, with 99% of these deaths occurring in low - and middle-income countries [3–6]. The rate of stillbirth in poor communities is likely to be two or more times greater than that in wealthier areas [18, 19]. The rate of stillbirths in UK was 4·2 per 1,000 [20], and Singapore and Finland had the lowest rates of stillbirths, at 2·0 per 1,000 [21]. However, in sub-Saharan Africa, it was 32·2 per 1,000 [15]. In our study, we found that the average stillbirth rate was 4.8 per 1000 in the last eight years and 2.3 per 1000 in 2022, was similar to what was observed in developed countries. However, we counted only antepartum stillbirths, the rate of stillbirths will be higher if we included intrapartum stillbirths and neonatal deaths. Given that the stillbirth rate was related to the state of the economy, the stillbirth rate was likely to be higher in less developed parts of China.

Stillbirths are very unfortunate events, and more worryingly, women who experienced a stillbirth are more likely to suffer the same outcome in later pregnancies [22]. Therefore, determining the cause of stillbirth is important and can help provide correct advice to parents about stillbirths and help them plan future pregnancies.

A meta-analysis and literature review revealed that primiparity was an important risk factor for stillbirth [23]. In our study, nearly 60% of stillbirths were primipara, which prompted us to pay attention to this issue. At the same time, we also found that 59.0% of stillbirths were from rural areas, which is consistent with the findings of previous studies [23, 24]. There is often a lack of health awareness and low socioeconomic status in most rural areas; therefore, women in these areas are more likely to experience stillbirth.

In our study, we found that the main causes of antepartum stillbirths were unspecified causes, antepartum hypoxia, and other specified antepartum disorder. However, in South Africa and the United Kingdom [7], the leading causes of antepartum stillbirths were unspecified causes, fetal anomalies and chromosomal abnormalities, and fetal death due to problems related to fetal growth.

Further detailed analysis of antepartum stillbirths revealed that 32.9% of antepartum stillbirths occurred before 28 weeks of gestation, which is basically consistent with the findings of Flenady [25]. While 51.2% of antepartum stillbirths occurred between 28 and 37 weeks of gestation, so this period is also worth considering.

In a Swedish study [26], it was found that causes of stillbirths vary with gestational age, we also found that the causes of stillbirths at different gestational ages were different. In addition to having unspecified causes, fetal anomalies and chromosomal abnormalities were more common before 28 weeks of gestation, antepartum hypoxia was the main cause between 28 and 37 weeks of gestation, and after 37 weeks of gestation. This finding prompted us to investigate whether further subdivide the antepartum stillborn births according to the ICD-PM system is necessary to obtain more accurate analysis results.

An accurate definition of the medical causes of stillbirths requires a minimum: (1) a complete obstetric record with frequent observations of maternal blood pressure, vaginal bleeding, and fetal heart rate; (2) a gross and histological placental examination; and (3) a fetal autopsy [27]. In our study, more than half of stillbirths were unexplained after review of clinical data and pathological examination of placenta. Autopsy is considered an ideal method for investigating the causes of perinatal deaths [25, 28]. Studies [29] have shown that in 22–76% of cases, autopsies can reveal new and valuable information. However, the autopsy acceptance rate in our study was only 3.6%, far lower than the level of western developed countries [30–32]. The main reason for refusing autopsies in our study was traditional concept of preserving the integrity of the body after death. Therefore, changing people’s traditional concept is critical to increasing the acceptance of autopsy.

However, McPherson emphasized that determining the exact cause of a baby’s death can be difficult even if an autopsy was performed [33]. Some studies [27, 34, 35] have suggested that CMA can help to determine the cause of stillbirth, but this may not be certain at present [36]. In our study, only 10.5% of parents received CMA, and 13.6% of them had abnormalities, suggesting that CMA may contribute to the identification of causes of stillbirths. The main reasons for rejecting CMA were a lack of understanding of CMA and high costs; thus, increasing the publicity of relevant knowledge and lowering the cost of the tests or incorporating them into medical insurance may help to increase acceptance of CMA.

## Conclusion

The ICD-10 is helpful in classifying the causes of stillbirths, but more than half of the stillbirths in our study were unexplained; therefore, additional work is needed. The ICD-10 score may need to be improved, such as by classifying stillborn patients according to gestational age. Regular obstetric examination is highly important for reducing the incidence of antepartum stillbirths. Autopsy and CMA could help to determine the causes of stillbirths, but their acceptance rates are currently low.

### Electronic supplementary material

Below is the link to the electronic supplementary material.

Supplementary Material 1

## Data Availability

All data are available from the corresponding author on reasonable request.

## Abbreviations

LMICs: Low and middle-income countries
ICD-10: International Classification of Diseases, tenth revision
ICD-PM: International Classification of Diseases for Perinatal Mortality
CMA: Chromosomal microarray analysis
BMI: Body mass index
