# Supplementary material for: The application of the ICD-10 for antepartum stillbirth patients in a referral centre of Eastern China: a retrospective study from 2015 to 2022
Source: BMC Pregnancy Childbirth. 2024 Feb 26;24:164. doi: 10.1186/s12884-024-06313-5 (PMC10895843; doi:10.1186/s12884-024-06313-5)
Supplement: Supplementary file 1 — Supplementary Material 1 [file 12884_2024_6313_MOESM1_ESM.docx]

**Changzhou Women and Children Health Hospital**

**Application form for medical ethics review**

| **Serial number** | | **2017003** | | | | **Project leader** | | | | **Bin Yu** | |
| --- | --- | --- | --- | --- | --- | --- | --- | --- | --- | --- | --- |
| **Project name** | | **Chromosomal microarray analysis** | | | | **Beginning and ending time** | | | | **From October 2016** | |
| **Item classification** | | | | | | **Application of new technology** | | | | | |
| **Brief Information of Applicants** | | | | | | | | | | | |
| **Name** | **Bin Yu** | | **Gender** | | **man** | **Doctor** | **University** | **Department** | | | **Laboratory** |
| **Telephone** | | | | **13861251515** | | **Email address** | | | **Ybcz0159@163.com** | | |
| **Current research direction** | | | | | | **Medical genetics** | | | | | |
| **Financial resources** | | | | | | **Other** | | | | | |
| **Item description:**  **Chromosomal microarray technology can be widely used in clinical practice. It can provide more effective genetic diagnosis for children with unexplained recurrent spontaneous abortion, stillbirth, and mental retardation, so as to guide clinical diagnosis and treatment.** | | | | | | | | | | | |
| **Promise of applicant:**  **All the above contents are true. If approved, I will carry out the project in strict accordance with the provided scheme and abide by the relevant regulations of the Ethics Committee of Changzhou Maternal and Child Health Hospital.**  **Signature: Bin Yu 2016-12-30** | | | | | | | | | | | |
| **Opinions of the leaders of the reporting unit:**  **I have reviewed this research project, and my organization has agreed to carry out this research.**  **Zhimao Cai 2017-06-01** | | | | | | | | | | | |
| **Opinions of the Medical Ethics Committee:**  **Consent**  **Jian Jiang 2017-06-01** | | | | | | | | | | | |
| **Conference No.** | | | | | | **201701** | | | | | |
| **Date of a conference** | | | | | | **2017-06-01** | | | | | |
| **Meeting place** | | | | | | **Conference room** | | | | | |
| **Conference chairman** | | | | | | **Jian Jiang** | | | | | |
| **Voting at the meeting** | | | | | | **signature** | | | | | |
| **Consent** | | | | | | **Jian Jiang Zhijun Shang Yishan Dong Jianguo Zhang** | | | | | |
| **Disagree** | | | | | | **No** | | | | | |
| **Waiver** | | | | | | **No** | | | | | |
